# Development and Validity Test of Social Attachment Multidimensional Scale

**DOI:** 10.3389/fpsyg.2021.757777

**Published:** 2022-01-04

**Authors:** Maosheng Yang, Kwanrat Suanpong, Athapol Ruangkanjanases, Wei Yu, Hongyu Xu

**Affiliations:** ^1^School of Economics and Management, Beijing Jiaotong University, Beijing, China; ^2^Chulalongkorn Business School, Chulalongkorn University, Bangkok, Thailand; ^3^School of Economics and Management, Jiangxi Science and Technology Normal University, Nanchang, China; ^4^Finance Office, Beijing Jiaotong University, Beijing, China

**Keywords:** attachment theory, social attachment, scale development, social media, reliability and validity

## Abstract

Social attachment can explain well the bond between users and social media, but existing research lacks measures of social attachment scales. To this end, this study takes attachment theory as the basis for scale development. On the basis of the development of multidimensional scales for adult, brand, and local attachment, it combines existing relevant studies on social attachment, selects three representative social media such as TikTok, WeChat, and MicroBlog as theoretical samples, explores the concept and structure of social attachment, and develops a social attachment scale through qualitative interviews and open-ended questionnaires. This study applied SPSS 24.0 and Mplus 7.0 to test the social attachment scale. The findings reveal that social attachment consists of three constructs: social connection, social dependence and social identity, and the scale possesses high reliability and validity. This study has developed and validated a social attachment scale in the context of social software use, realizing a quantitative study of social attachment and providing a basis for future empirical research related to social attachment.

## Introduction

In recent years, with the rapid development and in-depth application of the new round of information technology, as well as the comprehensive popularity and flourishing of local social media, a boom in theoretical research around the theme of attachment relationship between users and local social media has begun to emerge, becoming a valuable and theoretically constructive theme (Chen et al., 2020; Yang et al., 2021a). There is a growing awareness in business and academia that exploring social attachment can help companies acquire new ways of operating and growing, and gain and maintain a competitive advantage (Goel et al., 2011), and that generating social attachments may be the key to the persistent use behavior of local social media users in a virtual environment (Yang et al., 2021b). Social attachment has become a research trend in building emotional bonds between users and local social media.

Social attachment has become increasingly important in the field of virtual environments or information systems. Given that social media is a typical virtual environment and information system, relying on building emotional attachments between users and local social media is extremely important for social media platforms to maintain continuous usage in a highly competitive business environment (Zhao and Wang, 2016; Lemay et al., 2019). It is the emotional attachment that Facebook forms with its users that drives their continued use (Lin and Lu, 2011; Choi, 2013; Reinecke et al., 2014), gaining and maintaining a competitive advantage in business competition. It can be seen that emotional attachment is the main antecedent variable leading to the persistent use behavior of local social media users (Zhao et al., 2012).

Attachment has its origins in the field of mother–infant relationships and refers to a strong emotional bond formed between an individual (primarily the infant) and a specific object (the mother or caregiver; Bowlby, 1969). Bowlby (1969, 1982) argues that attachment is a basic human need that lasts throughout an individual’s life. As the theory has been applied in different fields, it has now been extended to other areas of adult relationships, focusing on the following themes according to the contexts in which attachment theory has been applied and developed. The first is brand attachment research. Brand attachment refers to the cognitive and emotional ties that consumers form with a brand, and the strength of these ties influence consumer behavior in a way that enhances brand profitability and lifetime customer value (Thomson et al., 2005; Fedorikhin et al., 2008). Second is place attachment research, first defined by Shumaker and Taylor (1983) as the emotional connection between people and their place of residence (Shumaker and Taylor, 1983). As research progressed, scholars generally agree with Hidalgo and Hernandez (2001) that place attachment refers to the emotional connection that is established between a person and a particular place to express the psychological state that people tend to stay in that place and feel comfortable and secure. Third is adult attachment research. Hazan and Shaver (1987) argue that romantic love between adults can also be seen as an attachment process, with similar bonding mechanisms to mother–infant attachment (Hazan and Shaver, 1987). The emotional connection to a partner in the attachment process can also be seen as an attachment relationship, i.e., adult attachment. The type of adult attachment influences the individual’s cognition, attitudes, and behavior during his or her development. Since then, adult attachment has become an important theoretical framework for research in the field of adult relationships (Hazan and Shaver, 1987). Fourth is social attachment research. Social attachment is an emotional and unique bond between users and social media (Yang et al., 2021a,b). Users with a strong sense of social media see social media as an integral part of themselves and are willing to devote extra personal resources such as time, energy and money to social media, resulting in continuous positive use behavior (Yang et al., 2021a).

Of the above studies, the first three start early and have produced a series of fruitful research results. For example, research on consumer–brand relationships and brand loyalty in the subject area of brand attachment research (Thomson, 2006; Vlachos et al., 2010; Malar et al., 2011; Japutra et al., 2014; Ilicic et al., 2016; Bidmon, 2017), etc. The study of place identity (Williams et al., 1992) and place dependency (Williams and Vaske, 2003) within the theme of place attachment research, etc. Among the themes of adult attachment research are parent–child attachment, marital attachment between lovers or spouses (Collins and Read, 1994) and peer attachment (Bretherton and Waters, 1985; Armsden, 1987). In contrast, the study of social attachment is relatively recent. Social attachment has become a research hotspot with the rise of the information technology revolution and attracted attention due to the continuous use behavior of information system users (Yang et al., 2021a). At the same time, the study of social attachment is at the forefront of research. Clearly, as a new concept, social attachment has not yet been described and measured in a procedural form, and its commercial practice and theoretical development need to be further explored (Yang et al., 2021b).

On the one hand, social attachment can be a good explanation for the bond between users and social media. It has been found that the willingness of users to build a lasting relationship with a particular social media and not move to other social media offering similar services or content is not only due to their lack of motivation or behavioral habits to switch, but also because they have an emotional attachment to that social media. On the other hand, existing research has explored the attachment relationship between users and social media to a limited extent. Quantitative research on social attachment is limited, with the biggest obstacle being the lack of research that has developed and tested social attachment scales (measurement tools) through a standardized process, leading to confusion about what social attachment entails. This raises new research questions about social attachment, how many dimensions of social attachment there are for local social media users, and what their content is. Furthermore, how does social attachment drive users’ continued use behavior. These questions need to be further researched and explored.

To this end, this study first summarizes and reviews existing attachment research, then develops an initial social attachment scale through interviews and open-ended questionnaires, follows a standardized procedure to further explore the concept and structure of social attachment, and conducts validity and reliability tests.

## Literature Review and Literature Review of Scale Development

### Attachment Theory

Attachment theory is derived from a study by Bowlby (1969) on parent–child relationships. In this study, Bowlby finds that infants form a unique emotional bond with their mothers or their caregivers and that this emotional bond influences the characteristics of the emotional bond and subsequent behavioral tendencies of the individual as he or she grows up (Bowlby, 1969, 2005; Levy, 2013). Therefore, Bowlby defines attachment as “a strong emotional bond between an individual and a specific object in their life” (Bowlby, 1982; Thomson, 2006; Liang and Wang, 2014). The relationship can be strong or weak, with a strong sense of attachment being associated with strong bonds, emotions, fondness, passion, etc. (Bowlby, 1982; Collins and Read, 1994). According to Bowlby (2005), the internal workings of attachment that develop in infants through ongoing interactions with their mothers or caregivers are indicative of “the quality of emotional bonds and the nature of psychological expectations between children and their mothers or caregivers and also have an impact on the self-concept and peer relationships they form later in life” (Ainsworth et al., 1978). Because the internal workings of attachment form the basis of other social relationships, early attachment relationships are of great importance in the emotional and social development of individuals throughout their lives and development (Crittenden, 2017; Kim and Wickrama, 2020).

Research has shown that attachment theory, as a unique emotionally charged bond between an individual and a specific object, has been applied to explain strong emotional, cognitive, and behavioral connections between individuals and specific objects (Bowlby, 1969; Hazan and Shaver, 1987). Hazan and Shaver's (1987) research further extends attachment theory from the realm of mother–infant relationships to other relationship domains, and it has received extensive attention, development and application in the areas of brand attachment (Thomson et al., 2005), place attachment (Shumaker and Taylor, 1983; Hidalgo and Hernandez, 2001), and adult attachment (Hazan and Shaver, 1987).

Based on the expansion and application of attachment theory in the brand attachment, place attachment, and adult attachment, this study defines “a strong emotional bond between users and social media” as social attachment, based on the integration of existing research on social attachment (Yang et al., 2021b). Social attachment can well explain the bond between users and social media, but existing research lacks measures of social attachment scales. Valid measurement of the construct is an important foundation for empirical research, and as a relatively new construct, social attachment has not yet been validly measured. Therefore, this study used Yang et al.’s (2021b) social attachment theory as a framework for scale development. First, the attachment between users and social media based on the level of cognitive representations is measured as a reflection of the characteristics of implicit or explicit associations formed by individuals based on past experiences, emotional responses, or cognitive representations. Second, the functional level of attachment between users and social media based on the physical environment is measured as a reflection of the properties that reflect the basic needs of users for a particular social media or a feature of social media. Finally, the spiritual-based attachment between users and social media is measured as a reflection of attributes that reflect the extent to which individuals view social media as self-presentation and identity.

### Adult Attachment and Its Measurement Methods

Adult attachment refers to the unique emotional bond between adults in a marital or parent–child attachment relationship (Hazan and Shaver, 1987, 1990; Buchheim et al., 2006). Unlike infant attachment, when people grow up, the objects of adult attachment increase and form a hierarchical system. However, Bowlby (1982) argues that the direction of adult attachment development is influenced by the “internal workings” of attachment formation in infancy. Thus, in the process of continuously selecting and adapting to the environment, the existing attachment system is consolidated and maintained, allowing for a certain continuity of attachment (Koh et al., 2020).

One of the more representative studies of the multidimensional measurement of adult attachment is the Experience of Intimacy Scale. The scale was originally developed by Brennan et al. (1998) based on item response theory, which divided attachment into two dimensions, anxiety and avoidance, and developed a two-dimensional self-report scale containing 36 items. It has been confirmed by subsequent scholars to have good reliability and validity and has been widely used and accepted (Noftle and Shaver, 2006). These two dimensions, anxiety and avoidance, provide a complete picture of what the attachment system is all about. According to Fraley et al. (2011), anxious ambivalence and avoidant attachment constructs are the two main subsystems of the attachment system in adult relationships; both constructs have a role in supporting the individual to maintain psychological and emotional proximity to the attachment partner in the attachment relationship. The role of anxious attachment is to enable the individual to judge whether the attachment partner is able to maintain constant psychological and emotional proximity to him or her and to be sensitive to the other person’s emotional responses, which can lead to anxiety or ambivalence if the attachment partner is not aware of the individual’s psychological or emotional needs. It can be seen that these two constructs, one focusing on judgments of the attachment object’s behavior and the other on judgments of one’s own behavior. If the individual is good at functioning as anxious, he or she will be busy constantly checking whether the other person loves him or her and worrying whether the other person will abandon him or her, but if the other side of the individual is also well developed in the avoidance dimension, he or she will be busy constantly avoiding closeness with the other person and negatively asking for attachment and emotional proximity. Therefore, Noftle and Shaver (2006) defines this high anxiety, high avoidance type as a disorganized attachment style, where the attachment relationship is in a state of both closeness and avoidance when the anxiety and avoidance characteristics are too pronounced, and vice versa, i.e., low anxiety and low avoidance means that these two characteristics are less pronounced and the love relationship has a secure attachment style (Karatas and Demir, 2019).

It is clear that adult attachment has become a focus of attention in recent years as it affects the formation of personality traits, the expression of interpersonal social functioning, coping styles, stress responses, psychological well-being, and morbidity (Ravitz et al., 2010; Umemura et al., 2017; Sommantico et al., 2019).

### Place Attachment and Its Measurement Method

Shumaker and Taylor (1983) defines place attachment as the emotional connection between people and their place of residence. Although this definition emphasizes the important role of emotions in the relationship between individuals and places, it is difficult to use to distinguish place attachment from other similar concepts (Morgan, 2010). The definition of the concept of place attachment has been the subject of much discussion by subsequent scholars, and although the measurement of the multiple dimensions of place attachment varies, there is general agreement that the core of place identity is the generic concept of ‘the emotional connection one forms to a particular place (Khosravi et al., 2020).

In terms of scales for measuring place attachment, academics generally agree that place attachment is a multidimensional construct, and a number of scholars have conducted research on it. A typical example is Williams et al.’s (1992) two-dimensional scale, which suggests that the structure of place attachment consists of two sub-dimensions, place attachment, and place identity. This theory has been positively responded to and further developed by subsequent scholars. Specifically, place dependence refers primarily to the ability of a particular place to satisfy the specific goals and functional needs of the user, indicating an individual’s preference for a particular place (Lewicka, 2011). Unlike place dependency, place identity emphasizes the spiritual dimension of the “human-place” connection and the emotional ties that one forms to a particular place based on direct or indirect experience. Place identity reflects not only an individual’s comprehensive feeling and evaluation of the real world in which he or she lives, but also the importance he or she places on the construction of the self, the presentation of the self as a unique emotion, and symbolic value (Anton and Lawrence, 2014).

Research has concluded that although the two-dimensional structure of place attachment has been widely adopted and its reliability in terms of reliability and validity has been demonstrated in a variety of contexts (Lee et al., 2012; Ramkissoon et al., 2012), it has also been noted that measuring place attachment using only the two-dimensional structure is difficult to convey its connotations, and therefore, the two-dimensional structure has been further developed into a three-dimensional or four-dimensional structure of place attachment (Ramkissoon et al., 2012; Brown et al., 2016). In the existing multidimensional research of place attachment, in addition to place identity and place dependence, the widely recognized new dimensions are mainly emotional connection (Cardinale et al., 2016; Jiang, 2017), social connection (Ramkissoon et al., 2012; Jiang, 2017), and natural connection (Strzelecka et al., 2017).

Undoubtedly, place attachment, as a measure of an individual’s relationship with a particular place, has become an important part of predicting people’s need for intimate emotional connections and stress-relieving experiences in a particular place (Cuni-Sanchez et al., 2019; Gao et al., 2019) and has become an important topic of academic research.

### Brand Attachment and Its Measurement Method

Brand attachment is an emotional bond and connection that individuals form with a brand based on the perceived image of the brand (Thomson et al., 2005). It reflects the emotional commitment and attachment that consumers form to a particular brand (Shimul et al., 2019), which expresses the degree to which the brand is “passionately” associated with the consumer’s self (Fastoso and Gonzalez-Jimenez, 2020).

Representative of multidimensional measures of brand attachment is the two-factor model and the three-factor model. The two-factor model suggests that brand attachment consists of two constructs: brand-self association and brand awareness (Fedorikhin et al., 2008). The brand-self connection is a central part of attachment and involves the emotional orientation of the brand in the consumer’s psychological experience. For example, when the brand is close to the consumer’s perception, it brings the consumer a happy and joyful experience, while when the brand is different from the consumer’s perception, it causes the consumer to feel anxious or sad. It can be seen that the brand-self connection reflects consumers’ personal aspirations (Mittal, 2006) and builds an emotional and cognitive connection between the brand and the consumer (Chaplin and John, 2005; Escalas and Bettman, 2005; Lam et al., 2011). Based on the brand-self connection, consumers see the brand as part of themselves and the psychological feelings and experiences built around the brand-self connection are further developed to create brand awareness. Brand awareness is the accumulation of ideas and feelings that individuals form about a brand and are constantly embedded and reinforced in consumers’ memories, reflecting the strength of the emotional bond between the brand and the consumer. In research on the multidimensional measurement of brand attachment, some studies have argued through scale development that brand attachment is a three-factor scale consisting of emotion, passion, and connection. The emotion factor is about deep affection, friendliness, love, and gentleness; the passion factor is about enthusiasm, elation, and infatuation; and the connection factor is about association, contract, and attachment (Thomson et al., 2005). In addition, Harrigan et al. (2018) further expands brand relationship to virtual environmental use situations on the basis of Fournier (1998) and found that individuals have self-related, partner quality, love and passion, and intimate feelings.

It is evident that along with the enrichment and development of the multidimensional scale of brand attachment, brand attachment plays an important role in the strategic management of companies, playing a central role in motivating and managing brand relationships in terms of user satisfaction, trust, and commitment (Wen et al., 2019). In marketing contexts, brand attachment becomes an important basis for influencing the strength of the emotional bond between individuals and brands, helping to foster the bond between individuals’ selves and brands and to enhance the automatic recall of brand feelings (Dwivedi et al., 2019), which in turn drives consumers to show higher levels of brand commitment. This helps to increase the allocation of resources to brand building (Yu, 2020), thereby enhancing brand profitability, fostering customer loyalty and gaining competitive advantage.

In summary, as attachment theory has evolved, more and more research has begun to explore the in-depth application and development of attachment in different domains. The definition and multidimensional measurement of new concepts such as brand attachment, place attachment, and virtual space attachment are all new developments and reconstructions of attachment theory in new contexts. According to Bagozzi (2006), “attachment is a promising and important topic worthy of research.” Zeithaml et al. (2006) also confirm that attachment theory can provide a guiding direction for customer relationship research, enriching the depth and breadth of existing research on attachment within the marketing field.

In view of this, this study uses attachment theory as the basis for scale development, and develops a multidimensional measure of social attachment with good psychometric properties for the specific relationship of “user-social media” in local social contexts. During the development of the scale, the development and conceptual definition of previous scholars’ multidimensional attachment scales in attachment contexts such as adult attachment, brand attachment, local attachment, and virtual space are fully considered in order to better profile the predictive power of the user’s perceptions and behaviors in specific relationship contexts.

## The Development Process of the Social Attachment Scale

Based on the analysis of existing literature on attachment theory, this study collects primary data on social attachment based on Chinese users’ perspectives through in-depth interviews and developed a social attachment scale based on rooting theory (Strauss and Corbin, 1990; Chen, 2015). This is because, on the one hand, the principles and methods of scale development by psychometric theory are to collect raw data through in-depth interviews and open-ended questionnaires, to systematically code, summarize and refine the raw data to form an initial scale and measurement items (Creswell, 2012), and to pre-test the initially formed measurement items to produce a formal questionnaire with good reliability and validity. It is a scientific method of scale development (Chen, 2000; Jin et al., 2016). On the other hand, this study is based on the strong emotional relationship between users and social media, i.e., the phenomenon of continuous use behavior selecting representative users as interviewees, and through deep mining of data with a view to discovering and revealing the concepts and constructs of social attachment, which is a typical process of generating from phenomena to theory, and is suitable for scale development using rooting theory (Charmaz, 2009; Zhang and Yang, 2018). The process of developing the social attachment scale in this study is as follows.

### Collection of Primary Information

Firstly, a review of existing research on social attachment is conducted to understand the concept and structure of social attachment. Secondly, the literature analysis software CiteSpace is used to search the relevant domestic and international literature and to analyze the existing methods of developing scales for attachment multidimensional variables. Drawing on the scale measurement paradigm of attachment theory within the scope of existing research hotspot areas, reference is made to the rationale for the development of multidimensional scales for brand attachment, local attachment, parent–child attachment and adult attachment. Again, in-depth interviews and focus groups were used to further capture the perceptions of social attachment based on the perspectives of local Chinese social media users. The interview outline focuses on the following three levels: general information on the use behavior of local social media users, including the behavioral habits, content used, and inner experience of using local social media; past experiences of using local social media; and the emotional relationship between users and the app in the process of using local social media. The general context is broader, with past experiences focusing on the act of using local social media itself, and emotional relationships aiming to draw out the theme of attachment relationships further. On this basis, this study conducted follow-up interviews with users of the above three social media software from March to December 2018, and as of December 10, 2018, a total of 27 users of the three social media were selected for data collection, and after categorizing and processing the interview transcripts of 27 users, the data were cleaned to form a word file, which became the source of developing the social attachment initial questionnaire imported as a source of material. The demographic characteristics of the in-depth interview sample are shown in Table 1.

**Table 1:** Demographic characteristics of in-depth interview samples.

Data sources	User	Gender	Age	Educational background	Profession	Cumulative use time
TikTok	TikTok users1	Male	25	Bachelor	Country startup	Over 3 years
	TikTok users2	Female	22	Bachelor	Clerk in private enterprise	1 ~ 2 years (inclusive)
	TikTok users3	Male	35	High school	e-commerce startup	6 months to 1 year (inclusive)
	TikTok users4	Female	27	Doctoral candidate	Student	1 ~ 2 years (inclusive)
	TikTok users5	Female	28	High school	Housewife	1 ~ 2 years (inclusive)
	TikTok users6	Female	30	Junior college	TikTok startup	1 ~ 2 years (inclusive)
	TikTok users7	Male	30	Junior college	Executive in private enterprise	1 ~ 2 years (inclusive)
	TikTok users8	Female	26	Bachelor	Sirhostess	1 ~ 2 years (inclusive)
	TikTok users9	Female	30	Bachelor	Startup	Over 3 years
	TikTok users10	Female	30	Junior college	Housewife	Over 3 years
	TikTok users11	Male	45	Junior college	Startup	1 ~ 2 years (inclusive)
MicroBlog	MicroBlog users1	Female	33	Bachelor	Senior high school teacher	Over 5 years
	MicroBlog users2	Male	26	Doctoral candidate	Student	Over 10 years
	MicroBlog users3	Female	26	Bachelor	Writer	Over 5 years
	MicroBlog users4	Female	20	Bachelor	Clerk in state-owned enterprise	Over 5 years
	MicroBlog users5	Female	23	Bachelor	Freelance	Over 5 years
	MicroBlog users6	Female	29	Junior college	Civil servant	7 ~ 10 years (inclusive)
	MicroBlog users7	Female	23	Bachelor	Freelance	7 ~ 10 years (inclusive)
WeChat	WeChat users1	Female	20	Bachelor	Editor	7 ~ 10 years (inclusive)
	WeChat users2	Male	35	Bachelor	Manager of private enterprise	Over 10 years
	WeChat users3	Male	35	Bachelor	Executive in private enterprise	Over 10 years
	WeChat users4	Male	37	Junior college	Startup	Over 10 years
	WeChat users5	Male	35	Doctoral candidate	College teacher	Over 10 years
	WeChat users6	Male	30	Junior college	Executive in private enterprise	7 ~ 10 years (inclusive)
	WeChat users7	Female	38	High school	Startup	Over 10 years
	WeChat users8	Male	40	Doctoral candidate	College teacher	7 ~ 10 years (inclusive)
	WeChat users9	Male	26	Junior college	Executive in private enterprise	7 ~ 10 years (inclusive)

### Development of the Initial Questionnaire

Based on the coding and landing of information collected through individual interviews and focus groups and the creation of attributions, multidimensional questionnaire items on social attachment were compiled as well as a frequency ranking. The lower frequency items were removed, resulting in an initial social attachment questionnaire consisting of 20 items. In order to test the consistency and rigor of the questionnaire determined by this method, 21 randomly selected mass users among the senior users of the above three major social media were tested between June 15 and July 30, 2018, and relevant experts were invited to review the initial questionnaire contents and modify the scale questions according to the actual situation of users’ social attachment. On this basis, the question statements were formulated to be as complete, written, and concise as possible. As the entries were written in a bottom-up manner based on the textual information from the interviews, the topics were not deleted and merged for the time being in this study. This resulted in 20 questions measuring social attachment based on the user’s perspective in the local Chinese context, as shown in Table 2.

**Table 2:** Analysis of interview contents.

Component dimension	Initial scale items
Social connection (SC)	Local social media (e.g., TikTok/WeChat/MicroBlog) has a special connection with me
	Local social media (e.g., TikTok/WeChat/MicroBlog) has become a part of my life
	Local social media (e.g., TikTok/WeChat/MicroBlog) works for self-presentation or emotional expression
	As a user of local social media (e.g., TikTok/WeChat/MicroBlog), I feel like part of its family.
	The use of local social media (e.g., TikTok/WeChat/MicroBlog) has brought me joy and happiness.
	Local social media (e.g., TikTok/WeChat/MicroBlog) has become a part of my work.
Social identity (SI)	Local social media (e.g., TikTok/WeChat/MicroBlog) means a lot to me.
	I really like local social media (e.g., TikTok/WeChat/MicroBlog).
	I strongly identify with local social media (e.g., TikTok/WeChat/MicroBlog).
	Local social media (e.g., TikTok/WeChat/MicroBlog) reflects my personality and style.
	I have no commitment to local social media (e.g., TikTok/WeChat/MicroBlog).
	I am immersed in local social media (e.g., TikTok/WeChat/MicroBlog).
	I have an obsession with local social media (e.g., TikTok/WeChat/MicroBlog).
Social dependence (SD)	I prefer this social media to similar social media (e.g., TikTok/WeChat/MicroBlog).
	I get more satisfaction from local social media (e.g., TikTok/WeChat/MicroBlog).
	Using local social media (e.g., TikTok/WeChat/MicroBlog) is more important than other (any) social media.
	There is no substitute for local social media (e.g., TikTok/WeChat/MicroBlog) compared to other (any) social media.
	TikTok/WeChat/MicroBlog are my favorite local social media.
	TikTok/WeChat/MicroBlog are the local social media I rely on the most.
	I would spend more time on local social media (e.g., TikTok/WeChat/MicroBlog) if I could.

On the basis of the content analysis of the social attachment interviews, three social media researchers in the field of marketing processed the initial social attachment measurement items formed from the interviews, focusing on the written expressions, semantic integrity and the overall contents of the component dimensions of each measurement item, and revised the items that did not meet the academic norms to ensure the content validity and surface validity of the measurement items; thus, the questions were revised to ensure the content validity and face validity, and then the social attachment questionnaire was developed, as shown in Table 3, to form the social attachment pre-test questionnaire.

**Table 3:** Measurement items of social attachment.

Component dimension	Item
Social connection, SC	1. TikTok/WeChat/MicroBlog have a special connection with me (e.g., star chasing, entertainment).
	2. TikTok/WeChat/MicroBlog have become a part of my life or work.
	3. TikTok/WeChat/MicroBlog works for self-expression or emotional expression.
	4. TikTok/WeChat/MicroBlog have brought me joy and happiness.
	5. TikTok/WeChat/MicroBlog can satisfy the need for virtual self-socialization.
	6. Local social media (e.g., TikTok/WeChat/MicroBlog) has become a part of my life or work.
Social dependence, SD	1. I prefer TikTok/WeChat/MicroBlog to similar social media.
	2. I get more satisfaction from TikTok/WeChat/MicroBlog.
	3. Using TikTok/WeChat/MicroBlog are more important than other (any) social media.
	4. There is no alternative to TikTok/WeChat/MicroBlog compared to other (any) social media.
	5. TikTok/WeChat/MicroBlog are my favorite local social media.
	6. TikTok/WeChat/MicroBlog are the local social media I rely on the most.
	7. I would spend more time on TikTok/WeChat/MicroBlog if I could.
Social identity, SI	1. TikTok/WeChat/MicroBlog means a lot to me.
	2. I really like TikTok/WeChat/MicroBlog.
	3. I am very attached to TikTok/WeChat/MicroBlog.
	4. TikTok/WeChat/MicroBlog can reflect my personality and style.
	5. I have no commitment to local social media (e.g., TikTok/WeChat/MicroBlog).
	6. I am immersed in local social media (e.g., TikTok/WeChat/MicroBlog).
	7. I have an obsession with local social media (e.g., TikTok/WeChat/MicroBlog).

### Social Attachment Scale Pre-test

The pre-test of this study was conducted with users of social media such as TikTok 50 online. Users were recruited as volunteers for this pre-test questionnaire, 50 copies were distributed, and 50 copies were collected, yielding 50 valid questionnaires, with a validity rate of 100%. Data collection was completed from October 25, 2018, to November 25, 2018. The following procedures were examined for the questionnaire items during the pre-test. The results of the social attachment scale pre-test are shown in Tables 4 and 5.

**Table 4:** Items analysis.

Item	Group (Mean ± Standard deviation)		*t*	*p*
	Low group (*n* = 23)	High group (*n* = 24)		
SC1	2.87 ± 1.25	5.08 ± 1.47	−5.538	0.000^**^
SC2	2.61 ± 1.47	5.42 ± 0.97	−7.754	0.000^**^
SC3	3.22 ± 1.13	5.42 ± 0.88	−7.475	0.000^**^
SC4	2.52 ± 1.04	4.92 ± 1.10	−7.667	0.000^**^
SC5	3.91 ± 1.47	5.92 ± 0.58	−6.077	0.000^**^
SC6	2.65 ± 1.07	5.17 ± 1.05	−8.13	0.000^**^
SD1	3.30 ± 1.06	5.58 ± 0.88	−8.018	0.000^**^
SD2	3.30 ± 1.26	5.13 ± 1.30	−4.882	0.000^**^
SD3	3.30 ± 1.22	5.25 ± 0.90	−6.241	0.000^**^
SD4	4.17 ± 1.99	5.17 ± 1.20	−2.057	0.047^*^
SD5	3.74 ± 1.29	5.33 ± 1.20	−4.388	0.000^**^
SD6	2.91 ± 1.12	5.33 ± 1.01	−7.779	0.000^**^
SD7	2.65 ± 1.15	5.25 ± 1.07	−8.001	0.000^**^
SI1	2.70 ± 1.22	4.96 ± 1.33	−6.054	0.000^**^
SI2	2.30 ± 1.02	5.46 ± 1.35	−9.005	0.000^**^
SI3	2.39 ± 1.16	5.00 ± 1.32	−7.195	0.000^**^
SI4	2.61 ± 1.27	5.54 ± 0.83	−9.32	0.000^**^
SI5	3.74 ± 1.66	5.75 ± 1.15	−4.811	0.000^**^
SI6	3.43 ± 0.95	5.88 ± 0.85	−9.314	0.000^**^
SI7	2.87 ± 1.14	5.71 ± 0.75	−10.036	0.000^**^

*SC is social connection, SD is social dependence, SI is social identity*.

* *p < 0.05*;

** *p < 0.01*.

**Table 5:** Reliability analysis.

Item	Item-total statistics	Corrected item-total correlation	Cronbach s alpha if item deleted	Cronbach α	Result
SC1	0.473^**^	0.606	0.827	0.848	Reserved
SC2	0.587^**^	0.684	0.812		Reserved
SC3	0.446^**^	0.631	0.823		Reserved
SC4	0.482^**^	0.66	0.817		Reserved
SC5	0.416^**^	0.547	0.838		Reserved
SC6	0.427^**^	0.257	0.817		Remove
SD1	0.339^**^	0.295	0.788	0.834	Remove
SD2	0.355^**^	0.646	0.797		Reserved
SD3	0.179^**^	0.659	0.794		Reserved
SD4	0.298^**^	0.362	0.844		Reserved
SD5	0.304^**^	0.501	0.819		Reserved
SD6	0.515^**^	0.606	0.802		Reserved
SD7	0.466^**^	0.695	0.788		Reserved
SI1	0.294^**^	0.561	0.847	0.859	Reserved
SI2	0.352^**^	0.633	0.839		Reserved
SI3	0.418^**^	0.267	0.832		Remove
SI4	0.242^*^	0.718	0.825		Reserved
SI5	0.443^**^	0.285	0.858		Remove
SI6	0.403^**^	0.652	0.836		Reserved
SI7	0.451^**^				Reserved

*SC is social connection, SD is social dependence, SI is social identity*.

* *p < 0.05*;

** *p < 0.01*.

First, the Pearson correlation coefficients of the questions are calculated and those with high correlation coefficients (above 0.9) are deleted or combined. The correlation coefficients of all the questions in this study were below 0.9. Therefore, all titled items are retained at this stage of the study. Second, the contents of the pre-test data are judged by item analysis, with the aim of statistically determining the relevance of the items and calculating the (CR) decision value for each item; the results of the data analysis showed that all items reach the significance level and are therefore retained. Third, the correlation between the product difference of each item and the total score is calculated and the item not reaching 0.3 is deleted. All other items are found to be significant and are therefore retained. Fourth, the Cronbach alpha coefficient and CITC values are analyzed. When the Cronbach alpha coefficient is above 0.8, it indicates high reliability; when the Cronbach alpha coefficient is between 0.7 and 0.8, it means good reliability; if this value is between 0.6 and 0.7, it indicates acceptable reliability; if the Cronbach alpha coefficient is less than 0.6, it indicates poor reliability. If the CITC value is below 0.3, the item may be considered for deletion. Therefore, SC6, SD1, SI3, and SI6 are excluded from this study, and the remaining questions are retained.

From the results of the pre-test analysis above, it can be understood that the pre-test questionnaire did not delete any items through item analysis, and then the reliability analysis revealed that the values of the indicators met the criteria for the questionnaire, and therefore, the pretest questionnaire could be used as a questionnaire for the formal survey. The final Social Attachment Questionnaire consisted of 16 items.

## Empirical Testing of the Social Attachment Scale

### Test Process and Subjects

In this study, the mass users of three local apps, TikTok, WeChat, and MicroBlog, are selected as the subjects of the study. The main reasons why these three are selected for data research in this study are as follows. First, the three brands belong to three different categories, with TikTok being a short music video social media, WeChat being a comprehensive social media and MicroBlog being a traditional social media, which has a wide distribution in terms of categories. Second, TikTok, WeChat, and MicroBlog are all iconic local apps and are leaders in their respective market segments. Therefore, these three apps have the typical characteristics required for scale development. Therefore, from this perspective, the use of TikTok, WeChat, and MicroBlog mass users to conduct data research is in line with the original research intention of this study. Again, this study used TikTok, WeChat, and MicroBlog as the samples for data collection. Although these three social media belong to different types of social media, the analysis of the data from the sample of users of different types of social media to draw commonalities in users’ social attachments helps to enhance the generalizability of the findings of this study. Therefore, the findings of this study are clearly representative. The questionnaire for this study consisted of 16 items, all on a 7-point Likert scale, with levels of agreement ranging from “strongly disagree” to “strongly agree,” with scores of 1 to 7. This study was conducted with questionnaires distributed on the paid platform of Questionnaire Star and completed from October 9 to December 20, 2018. A total of 520 questionnaires were distributed, 452 were returned, and 376 valid questionnaires were obtained after excluding invalid questionnaires, and the effective rate of the questionnaire survey was 83.19%. The specific characteristics of the sample are shown in Table 6.

**Table 6:** Analysis of basic data of samples.

Variables	Item	Frequency	%	Cumulative %
Gender	Male	160	42.55	41.28
	Female	216	57.45	100
Age	Under 20 years old (inclusive)	135	35.90	35.90
	21 ~ 25 years (inclusive)	102	27.13	63.03
	26 ~ 30 years (inclusive)	92	24.47	87.50
	31 ~ 40 years (inclusive)	35	9.58	97.08
	41 ~ 50 years (inclusive)	9	2.39	99.47
	51 years old and above	2	0.53	100
Marriage	Married (no children)	80	21.28	21.28
	Married (with children)	99	26.33	47.61
	Unmarried	172	45.74	93.35
	Divorced	25	6.65	100
Profession	Civil Servants	38	10.11	10.11
	Employees of state-owned enterprises	35	9.31	19.42
	Employees of private enterprises	108	28.72	48.14
	Employees of join-invested enterprises	37	9.84	57.98
	Students	81	21.54	79.52
	Other	77	20.48	100
	High school and below	60	15.96	21.51
	Bachelor	130	34.57	86.05
	Postgraduate and above	186	49.47	100
Consumption level	Under $1,500	119	31.0.65	31.98
	RMB1,500 ~ 1,999	126	33.51	59.88
	RMB 2,000 ~ 2,999	71	18.89	85.47
	RMB3000 and up	60	15.96	100
	6 months to 1 year (inclusive)	102	27.13	27.13
	1 ~ 2 years (inclusive)	124	32.98	60.11
	2 ~ 3 years (inclusive)	130	34.57	94.68
	3 ~ 5 years (inclusive)	20	32	100

This study uses SPSS 24.0 and Mplus 7.0 software for statistical analysis of the data. First, the items are analyzed with all valid data, and topics that are not discriminating are removed to ensure that the topics are discriminating. Second, all data are divided into two halves according to a randomized splitting method, with half of the data subjected to exploratory factor analysis and the other half subjected to validation factor analysis. The overall data are then used to test for reliability and validity. The results of the factor analysis are used to examine the multidimensional aspects of social attachment and to verify the reliability and validity of the scale.

### Items Analysis

To ensure that the developed questionnaire questions are valid, discriminatory analysis is a very important task in scale development. Therefore, a pre-test is conducted of the data using SPSS 24.0 on the sample collected. The aim is to confirm the semantic fluency of the scale questions, the absence of typos and the appropriateness of the layout. One of the most important tasks is to do an items analysis. Its purpose is to remove topics (or variables) that are not discriminating and use them as a basis for topic improvement.

Item analysis is essentially a t-test, which will check whether high and low subgroups are different. In this study, the dimensional items of social attachment were ranked from high to low. The data for all items were then divided into high, low, and medium clusters. Research has shown that there should be a significant difference between the mean of the data for the high and low scoring groups. If there is no significant difference, it means that the scores for the high scoring group are too close to the low scoring group, which means that this is an invalid questionnaire as there is no gap in the scores for all the questions. The results of the items analysis show that this is an invalid item, so it should be removed from this study (Liden and Maslyn, 1998; Babbie, 2004).

The specific approach and steps of the items analysis in this study are as follows. In the first step, the questions for each dimension are summed separately and transformed to calculate the new variable. In the second step, the values of the 27th and 73rd quartiles of each dimension are found. In the third step, the data for each dimension are divided into two groups: low and high. In the fourth step, each dimension is tested for significant differences between the high and low group means to determine whether the questionnaire questions are discriminatory. As can be seen in Table 7, the mean *t*-test *p* < 0.05, which represents a significant difference between the high scoring group and the low scoring group. Therefore, these items are to be retained in the study for their entirety.

**Table 7:** Analysis of scale items.

Item	Critical ratio	Item-total statistics	Result	Item	Critical ratio	Item-total statistics	Result
SC1	12.19^*^	0.93*	Reserved	SI4	11.22^*^	0.89^*^	Reserved
SC2	11.59^*^	0.95^*^	Reserved	SI5	11.01^*^	0.98^*^	Reserved
SC3	11.47^*^	0.96^*^	Reserved	SD1	10.61^*^	0.98^*^	Reserved
SC4	18.45^*^	0.95^*^	Reserved	SD2	13.03^*^	0.96^*^	Reserved
SC5	10.83^*^	0.98^*^	Reserved	SD3	17.45^*^	0.96^*^	Reserved
SI1	10.04^*^	0.92^*^	Reserved	SD4	11.23^*^	0.92^*^	Reserved
SI2	16.08^*^	0.91^*^	Reserved	SD5	13.70^*^	0.96^*^	Reserved
SI3	20.27^*^	0.87^*^	Reserved	SD6	14.74^*^	0.97^*^	Reserved

*SC is social connection, SD is social dependence, SI is social identity*.

* *p < 0.05*.

### Exploratory Factor Analysis

An exploratory factor analysis is conducted on the 16 items retained for item analysis using the data from the 188 sample items drawn from Part I to initially test the reliability of the multiple dimensions distilled during the development of the social attachment scale. The results of the analysis of the data according to SPSS 24.0 software show that the social attachment multidimensional scale has a KMO coefficient value of 0.925 and a Bartlett’s spherical test coefficient of 2365.59 (df = 159, *p* < 0.001), and the data indicate suitability for factor analysis. The analysis results show that there exists the possibility of sharing factors among the items; that is, there is a significant correlation between the variables. Therefore, it is appropriate to do an exploratory factor analysis on the sample data.

In this study, an exploratory factor analysis is conducted using the principal component method, and factor rotation is performed using the orthogonal rotation method to extract factors with eigenvalues greater than 1. A total of three factors are extracted in conjunction with the gravel plot test. The questions for the factors are removed based on the factor loadings. Where factor loadings greater than 0.35 are acceptable, ideally, they should be greater than 0.40 (Hinkin, 2005). Of course, it has been suggested (Lederer and Sethi, 1991) that questions with factor loadings below 0.35 should be removed, as less than 0.35 indicates a lack of reliability due to excessive measurement error. Previous scholars (Ding and Zhang, 2010) have even pointed out that a factor structure with a standardized factor loading (Standardized Factor Loading) of at least greater than 0.40 for all conformational items, without multiple loadings, and with roughly equivalent categorization of factor items, retaining important elements of relevant literature and interviews, is indicative of a good factor categorisztion.

In this study, the items are further screened according to the distribution of each indicator across the three factors, as suggested above, and the factor analysis is re-run each time the question items are removed, resulting in a factor loading of greater than 0.40 for each item, with no double loading. The final factor analysis is shown in Table 8, with a cumulative variance contribution of 55.07% for the three factors. This indicates that the factors are appropriately categorized and relatively satisfactory.

**Table 8:** Factor structure and factor loading of social attachment.

Measurement item	Construct		
	1. Social connection	2. Social dependence	3. Social identity
1. TikTok/WeChat/MicroBlog has a special connection with me (e.g., star chasing, entertainment).	0.721		
2. TikTok/WeChat/MicroBlog has become a part of my life or work.	0.647		
3. TikTok/WeChat/MicroBlog works for self-expression or emotional expression.	0.613		
4. TikTok/WeChat/MicroBlog has brought me joy and happiness.	0.697		
5. TikTok/WeChat/MicroBlog can satisfy the need for virtual self-socialization.	0.590		
1. I prefer TikTok/WeChat/MicroBlog to similar social media.		0.773	
2. I get more satisfaction from TikTok/WeChat/MicroBlog.		0.562	
3. Using TikTok/WeChat/MicroBlog is more important than other (any) social media.		0.850	
4. There is no alternative to TikTok/WeChat/MicroBlog compared to other (any) social media.		0.743	
5. TikTok/WeChat/MicroBlog are my favorite local social media.		0.784	
6. I would spend more time on TikTok/WeChat/MicroBlog if I could.		0.490	
1. TikTok/WeChat/MicroBlog means a lot to me.			0.702
2. I really like TikTok/WeChat/MicroBlog.			0.576
3. I am very attached to TikTok/WeChat/MicroBlog.			0.528
4. TikTok/WeChat/ MicroBlog can reflect my personality and style.			0.504
5. I have no commitment to local social media (e.g., TikTok/WeChat/MicroBlog).			0.785
Factor variance contribution rate	15.68	18.21	21.8

*Extraction method: principal component analysis. Rotation method: Varimax method with Kaiser normalization*.

The results of exploratory factor analysis show that the structure of social attachment includes three dimensions. Based on the meaning expressed by each factor item, this study has named them social connection, social identity, and social dependence. Specifically, the first factor is social connection, including five items. Social connection refers to the sense of membership that individuals form toward a group of people (e.g., friends in WeChat’s circle of friends, people they follow on MicroBlog’s homepage, fans on the TikTok platform, etc.) and the emotional bonds formed based on shared experiences, concerns, and interests (Yang et al., 2021a). Social connections are based on attachment at the level of cognitive representations and reflect the characteristics of implicit or explicit connections formed by individuals based on past experiences, emotional responses, or cognitive representations. The second factor is social identity, including five items. Social identity is a kind of spiritual attachment, which is used to explain the virtual identity of users and the social media use environment. It shows the emotional bond formed by the user based on the direct or indirect use experience and the social media use context of the specific virtual environment (Yang et al., 2021a). Social identity is based on spiritual attachment, which reflects the attributes that individuals regard social media as self-expression and identity (Yang et al., 2021a). The third factor is social dependence, including six items. Social dependence is the suitability of a specific virtual environment to meet individual functional needs and specific goals, reflecting the individual’s preference for ideal social media (Yang et al., 2021a). Social dependence is the attachment based on the functional level of the physical environment, reflecting the user’s attribute to a certain social media or the functionality of a certain feature of social media to meet the user’s basic needs. According to Hinkin (2005), the scale is best developed by keeping the number of items per dimension at four to six. Therefore, the social attachment scale developed in this study is reasonable in terms of the number of items.

### Confirmatory Factor Analysis

To further validate the stability of the three-factor structure of social attachment and to determine whether the results of the exploratory factor analysis could be supported by another sample, in this study, the second part of 188 sample data is used to conduct confirmatory factor analysis in order to verify the validity of the three-dimensional structure of social attachment. The results of the confirmatory factor analysis by applying Mplus 7.0 are shown in Table 9.

**Table 9:** Confirmatory factor analysis.

Construct	Item	Unstd.	S.E.	C.R.	P	STD.	SMC	CR	AVE
Social connection	SC1	1.00				0.68	0.46	0.91	0.758
	SC2	1.63	0.12	13.51	***	0.86	0.74		
	SC3	1.34	0.10	12.91	***	0.82	0.67		
	SC3	1.46	0.14	10.51	***	0.65	0.42		
	SC5	1.58	0.13	12.34	***	0.78	0.61		
Social dependence	SD1	1.00				0.73	0.53	0.87	0.54
	SD2	0.79	0.08	10.43	***	0.63	0.40		
	SD3	0.80	0.08	10.39	***	0.63	0.40		
	SD4	1.34	0.10	13.73	***	0.84	0.71		
	SD5	1.46	0.12	12.43	***	0.75	0.56		
	SD6	1.05	0.08	13.12	***	0.80	0.64		
Social identity	SI1	1.00				0.67	0.45	0.90	0.654
	SI2	0.95	0.10	9.83	***	0.61	0.37		
	SI3	1.35	0.10	13.74	***	0.90	0.81		
	SI4	1.36	0.10	14.02	***	0.92	0.85		
	SI5	1.40	0.10	13.59	***	0.89	0.79		

*SC is social connection, SD is social dependence, SI is social identity*.

*** *p < 0.001*.

Based on the criteria of Anderson and Gerbing (1988), this study selects unstandardized factor loadings, standard errors, standardized factor loadings, squared multivariate correlations, combined reliability and mean extracted variance for confirmatory factor analysis of all the constructs, and the results are shown in Table 9. According to the criteria proposed by Fornell and Lacker (1981), Nunnally and Bernstein (1994), and Hair et al. (1998) for convergent validity: (1) standardized factor loading of each indicator variable is higher than 0.50; (2) composite reliability is higher than 0.60; (3) average variance extracted is higher than 0.50. All the constructs in this study, standardized factor loading, ranged from 0.63 to 0.92, all of which are within the range, showing that each topic has topic reliability. The composite reliability of the study constructs range from 0.87 to 0.91, all of which exceed 0.7, all of which meet the criteria suggested by scholars, showing that each construct has good internal consistency. Finally, average variance extracted range from 0.54 to 0.76, all above 0.5, all meeting the criteria of Fornell and Lacker (1981) and Hair et al. (1998) showing that each construct has good reliability and convergent validity.

Model fit is analyzed using Jackson et al.’s (2009) recommendations for MLχ^2^, degrees of freedom (DF), Normed Chi-sqr (χ^2^/DF), RMSEA, SRMR, TLI (NNFI), CFI, GFI and AGFI (Janda, 1998; Kline, 2011), where smaller MLχ^2^ is better, larger DF is better, and 1 < Normed Chi-Square (χ^2^/DF) < 3, RMSEA < 0.08, SRMR < 0.08, TLI (NNFI) > 0.9, CFI > 0.9, GFI > 0.9, AGFI > 0.9,. where smaller MLχ^2^ is better and larger DF is better, while 1 < Normed Chi-sqr (χ^2^/DF) < 3, RMSEA < 0.08, SRMR < 0.08, TLI (NNFI) > 0.9, CFI comparative fit index > 0.9, GFI > 0.9, and AGFI > 0.9 indicating a good fit to the model (Bollen and Stine,, 1992; Hu and Bentler, 1999). The results of this study’s model fit are shown in Table 10, with all nine fit indicators reaching above an acceptable level, indicating a good fit for this study’s structural model.

**Table 10:** Indicators of model fit.

Model fit	Criteria	Model fit of research model	Result
Chi-square		678.76	
Degree of freedom		478	
CFI	>0.9	0.97	Acceptable
RMSEA	<0.08	0.04	Acceptable
TLI	>0.9	0.96	Acceptable
GFI	>0.9	0.92	Acceptable
NFI	>0.9	0.91	Acceptable
χ^2^/df	<3	1.42	Acceptable
AGFI	>0.8	0.89	Acceptable

### Reliability and Validity Analysis

#### Reliability Analysis

Crobanch’s alpha coefficient is applied to test the reliability of the internal consistency of the constructs and to verify the consistency of the items with each other, thus ensuring the reliability of the measurement questionnaire. Previous scholars have argued that if the value of Crobanch’s alpha coefficient for a factor is very low, it indicates that subjects have rather inconsistent attitudes toward the expectations of these items and should be removed. Based on the criteria used in previous exploratory studies in scale development, the following reliability tests for consistency of questions within the social dependence measurement instrument are used in this study: the internal consistency coefficient of Crobanch’s α within each factor is greater than 0.60 before it is retained, preferably above 0.70; at the same time, if the deletion of an item within the same construct leads to an increase in the Crobanch’s α coefficient for that construct, the item should be deleted rather than retained.

As shown in Table 11, the results of the reliability analysis of the three factors in this study reveal that the Crobanch’s alpha coefficients for social connectedness, social identity, and social dependence are 0.82, 0.75, and 0.93, respectively, for the three factors in this study, and the resulting internal consistency reliability coefficients range from 0.75 to 0.93, all of which are within the range, indicating that each topic has topic reliability. At the same time, there is no increase in the reliability of the construct when a topic is removed, and the internal consistency of all three factors of social attachment is greater than 0.70, indicating that the reliability of the social attachment measurement instrument scale is good.

**Table 11:** Reliability analysis of research variables.

Construct	Mean	Standard deviation	Crobach’s α
Social connection	6.20	0.89	0.82
Social identity	5.29	1.23	0.75
Social dependence	5.92	1.08	0.93

#### Validity Analysis

Validity analysis is the process of testing the validity and accuracy of measurement items. Content validity is a test to measure the applicability and representativeness of measurement items, that is, whether the measurement items truly reflect the concept that needs to be measured.

The Social Attachment Scale for this study is a measurement tool based on a local user perspective. In order to make the scale opening conform to the norms, in-depth interviews, semi-structured interviews, and focus group interviews with users through a standardized procedure are conducted, and the initial material from the interviews are coded level by level based on rooting theory to refine the initial questionnaire on social attachment. The whole process is carried out under the guidance of a PhD supervisor in business management, with the participation of two PhD students and two young faculty members specializing in integrated research methods (Warren et al., 2019). After several rounds of discussion, it has concluded that social attachment consists of three dimensions: social connection, social identity, and social dependence. The questions on the three dimensions of social attachment are developed through a rigorous process of refining the interview contents. It is evident that the social attachment scale has been developed in a scientific and rigorous manner. Therefore, the social attachment scale in this study has good content validity.

## Discussion and Conclusion

### Research Results

#### Reliability and Validity of Social Attachment

Based on a review of previous literature and qualitative interviews with social media users, this study extends the application of attachment theory to social media use contexts and develops and validates a measurement scale for social attachment. The development and testing of the Social Attachment Multidimensional Scale is carried out in strict accordance with standardized procedures and principles, resulting in a formal measurement scale with the three dimensions as its core contents, which is pre-tested and formally tested to obtain good reliability and validity. The results of the exploratory factor analysis show that the theoretical structure of the Social Attachment Scale is a good fit for the actual data obtained; individuals’ attachments to social media in the context of social media use are mainly in the areas of social bonding, social dependence, and social identity. The results of the exploratory factor test also indicate that there are significant differences in content and structure between the three dimensions of the Social Attachment Scale, reflecting the fact that social attachment is a concept and structure consisting of three different dimensions: social bonding, social dependence, and social identity. The results of the social attachment scale reliability tests further indicated that the Cronbach alpha values for the social connection, social identity, and social dependence scales are all above 0.70 in the acceptable range, indicating that the instrument has good internal consistency and that the social attachment construct has some stability and reliability (Fornell and Lacker, 1981; Hair et al., 1998) and can be measured quantitatively well. In addition, the measures of the latent variables in this study are based on a review of the existing attachment theory literature, the existing measurement paradigms in different domains such as brand attachment, local attachment and adult attachment, and the results of qualitative interviews and pre-studies to refine a multidimensional measure of social attachment. It is evident that the scale development process is scientifically rigorous and the measurement has good content validity. In conclusion, the reliability and validity measures indicate that there are good reliability and validity issues with the multidimensional scale of social attachment developed in this study, which provides a basis for subsequent quantitative research on social attachment.

#### Patterns of Social Attachment

This study taps into research on the bond between users and social media, delving into the differences in outcomes that arise from different attachment patterns. Existing studies on the continuous use of social media users mainly focus on the discussion of relatively limited antecedent variables such as perception, satisfaction, and loyalty (Choi, 2015). In addition, a single-dimensional perspective is used to analyze the predictive effect of users on the continuous use of social media. However, the change from need satisfaction to attachment formation to continued willingness of use behavior is often due to users forming emotional attachments to social media in some way, resulting in continued willingness and usage (Goel et al., 2011). In addition, although there is a wealth of theoretical research on personal attachment, there has been limited exploration of social attachment among social media users. Given the differences in traditional culture, habits, and laws, it is important to examine users’ persistent use behavior from a theoretical perspective and to provide references and suggestions for the business practice of social media. Therefore, this study compares the differences in outcomes produced by different social attachment patterns when users become emotionally attached to social media in terms of three dimensions of social attachment, using intention to continue using and as outcome variables. According to the results of this study, social attachment contains a total of three constructs, and the emotional bond between users and social media is closely influenced by three main aspects: social connection, social identity, and social dependence. The cumulative explainable variance of the three factors of social attachment is 55.07%, with the social connection factor explaining 15.68% of the variance, the social dependence factor explaining 18.21% of the variance, and the social identity factor explaining 21.18% of the variance. This suggests that social identity plays the most important role in users’ connotations of social media, followed by social dependency, with the social connection factor playing a relatively minor role as social connection is a psychological level of emotional bonding.

### Theoretical Contributions

#### Developing and Validating the Social Attachment Scale

Based on the analysis of existing literature on attachment theory, this study collects data through interviews and open-ended questionnaires under the guidance of rooted theory and uses a standardized procedure to develop the Social Attachment Scale. As the social attachment scale is developed based on bottom-up coding of the original interview material, there is a clear pathway for the formation of each question item, category, and relationship, which is strictly derived from the analysis of the interview data and can be found in the original information provided by the users, thus ensuring the integrity and clarity of the structured scales of the social attachment scale. There is no previous quantitative research on social attachment, leading to continued confusion about the fundamental question of what social attachment entails. Therefore, this study explores the concept and structure of social attachment through a normative procedure and conducts validity and reliability tests. It can be seen that this study not only develops a social attachment scale based on the perspective of Chinese users, but also bridges the gap in the previous literature on the mechanism of the role of social media use behavior.

#### Uncovering the Mechanisms at Play in the Attachment Relationship Between Users and Social Media

In previous studies on social media use contexts, scholars have not measured the construct of social attachment effectively. By drawing on the commonalities in the development of multidimensional measurement scales for adult attachment, local attachment, and brand attachment, this paper extends the application of attachment theory to social media use contexts and develops a multidimensional measurement scale for social attachment by combining qualitative interviews with social media users and collecting data from Chinese social media users to better profile the predictive power of users’ perceptions and behaviors in social media use contexts. This provides not only a concrete extension and application of attachment theory from the physical environment to the specific virtual environment of social media, but also a new perspective and useful reference for research on the attachment relationship between users and social media and a basis for future empirical research related to social attachment.

#### Adding New Explanatory Variables for Use Behavior of Local Social Media Users

Existing research on persistent social media use behavior has focused on a very limited number of antecedent variables such as perceived feelings, satisfaction, and loyalty (Choi, 2015), and has mostly analyzed the predictive role of users on persistent social media use from a univariate perspective. This study investigates the persistence mechanism of the relationship between users and social media from the perspective of emotional attachment, pointing out that social attachment is formed between users and social media, from need satisfaction to attachment formation to changes in behavioral intentions to continue using, leading to persistent use (Goel et al., 2011). Therefore, this study provides a useful addition to the existing studies which are mostly conducted from a transactional perspective and lack of explanatory power and provides a new research direction and explanatory variables for users’ continuous use behavior, thus providing a theoretical reference to further explore the relationship between social attachment the use of local social media.

### Practical Implications

#### Providing Actionable Tools for Social Media to Enhance Users’ Sustained Use Behavior

Currently, in the field of marketing management practices, more and more short video APPs are focusing on building continuous use behavior, hoping to differentiate their marketing strategies by building social attachments to create a competitive social media presence. However, all these efforts lack yet a theoretical and operationalizable instrument that can be implemented. The system of social attachment measurement instruments developed in this study distinguishes three dimensions of its composition. Of these, social identity explains the greatest variance, suggesting that social media developers should particularly strengthen the crafting of social identity when creating attachments between users and social media. Specifically, social media developers can provide users with a better experience through virtual platforms, making them a symbol of personal identity and making them a space for individual self-presentation and even identity, with the aim of winning a competitive advantage in business competition. On the one hand, it is important to focus on users’ past experiences, emotional reactions, or cognitive representations, so as to lead them to form implicit or explicit associations with social media; on the other hand, it is important to focus on users’ functional needs for social media or a particular feature of social media, so as to enhance their reliance on social media.

#### Providing Guidance for Developing a Strategy for Social Media Use

Understanding the multidimensional nature of social attachment is an important guide to building strong relationships with users in social media. Previous loyalty-guided marketing practice strategies are ineffective in motivating individuals to continue using and behaving in a consistent manner, resulting in a lack of targeted marketing strategies for social media platforms. This study examines the multidimensional concept of social attachment and finds that social attachment, as a relatively new construct, provides a valid core variable for social media to build ongoing user relationships. Social attachment helps social media to understand individuals’ consumption psychology and decision-making process, which leads social media to pay more attention to and work on building individuals’ emotional needs. Therefore, understanding and deconstructing the multidimensional characteristics of social attachment can help social media track and measure the level of social attachment of users, in order to understand the state of emotional connection between users and social media, grasp the relationship needs, expectations, preferences, willingness, and behavior patterns of users, and adopt appropriate marketing strategies to provide users with deep rational and emotional satisfaction, and stimulate and sustain their willingness and behavior to use social media. The marketing strategy is designed to provide users with deep rational and emotional satisfaction, inspiring and sustaining their continued use and behavior.

#### Providing Guidance for Building Social Attachments

In practice, building quality attachments with users is far more likely to bring sustainable returns to social media than an emphasis on rational analysis. On the one hand, users’ social attachment has a strong explanatory validity for continued usage intentions, implying that social media can win users’ continued use behavior by providing them with good product and service values in practice and by gaining insight into and understanding their emotions and spirit. On the other hand, perceived value, as an important antecedent influence on social attachment, helps guide social media to invest in key drivers that can build an emotional connection with users, thus helping social media to develop effective user retention strategies. Therefore, the emphasis on attachment helps guide social media to observe the strength of the emotional bond between users and social media in order to proactively adopt differentiated marketing strategies.

## Data Availability Statement

The original contributions presented in the study are included in the article/supplementary material, further inquiries can be directed to the corresponding authors.

## Author Contributions

MY, KS, and AR: conceptualization and writing original draft. MY and WY: data curation. MY and AR: formal analysis. MY, WY, and HX: investigation. MY, AR, KS, and HX: writing – review and editing. All authors contributed to the article and approved the submitted version.

## Conflict of Interest

The authors declare that the research was conducted in the absence of any commercial or financial relationships that could be construed as a potential conflict of interest.

## Publisher’s Note

All claims expressed in this article are solely those of the authors and do not necessarily represent those of their affiliated organizations, or those of the publisher, the editors and the reviewers. Any product that may be evaluated in this article, or claim that may be made by its manufacturer, is not guaranteed or endorsed by the publisher.
